# Pyloric Gland Adenoma of Gallbladder: A Review of Diagnosis and Management

**DOI:** 10.1155/2018/7539694

**Published:** 2018-12-19

**Authors:** Farid Saei Hamedani, Monica Garcia-Buitrago

**Affiliations:** ^1^Fellow of Gastrointestinal and Hepatobiliary Pathology, Jackson Memorial Hospital, Miami, FL, USA; ^2^Professor of Pathology, Director of Gastrointestinal Pathology Service, University of Miami Miller School of Medicine, 1611 NW 12 Ave Holtz Bldg. Room 2042, Miami, FL, USA

## Abstract

Neoplastic polypoid mucosal lesions of the gallbladder are increasingly being reported in cholecystectomy specimens. However, due to the absence of unified terminology and reporting criteria, the body of scientific evidence on their classification, prognosis, and management is scarce and sometimes controversial. While they have different histomorphologic features (gastric foveolar, gastric pyloric gland, biliary, and intestinal), a significant immunohistochemical overlap exists which highlights their mixed cell lineage with a dominant cell type in each, establishing the subcategory. Because of many shared attributes, intracholecystic papillary-tubular neoplasm (ICPN) has been introduced as an umbrella terminology. ICPNs of the pyloric subtype are lesions larger than 1 cm, as most of the smaller ones are clinically insignificant and represent polypoid hyperplasia rather than a true neoplasm. In this review, we will focus on the pyloric gland adenomas as the most frequent histologic subtype of ICPNs.

## 1. Introduction

Neoplastic polyps of the gallbladder are commonly asymptomatic [1]. However, advances in radiologic modalities and their growing use for various clinical indications have increased the number of gallbladder polyps being diagnosed and reported [2]. Yet, due to lack of unified terminology and reporting criteria, the body of scientific evidence regarding their classification and management is scarce and even sometimes controversial [3]. The plethora of terminology used in scientific literature to describe these lesions includes “pyloric gland adenoma,” “tubulopapillary adenoma,” and “biliary adenoma” [3]. Even though this diverse group of lesions shares histological and immunohistochemical characteristics, they are distinct entities with different cellular lineages and a spectrum of dysplasia which makes their prognosis different. Histologically, these lesions are classified as the gastric pyloric gland, gastric foveolar, intestinal, and biliary [4], with the pyloric subtype being the most common lesion (82%) [4]. Adsay et al. are the first group of investigators who proposed the unified terminology of intracholecystic papillary-tubular neoplasms (ICPNs) to describe neoplastic polyps of the gallbladder [3]. They used the size of over 1 cm as an inclusion criterion as this size has been used in other lesions of the pancreatobiliary system like intraductal papillary mucinous neoplasms (IPMN). In the surgical literature, patients with polyps of over 1 cm are often being elected to go through cholecystectomies [5]. Adsay and colleagues used 25% and 75% tubule or papillary formation as cutoff points to categorize ICPNs based on their growth patterns, and so 43% of their cohort qualified as papillary, 26% as tubular, and 31% as tubulopapillary. The mean sizes of the papillary, tubulopapillary, and tubular polyps were reported as 2.8 cm, 2.7 cm, and 2 cm, respectively [3]. It is explainable, as in other parts of the gastrointestinal tract, smaller lesions are usually more tubular and papillary lesions are often larger [3]. They reported the biliary type as the most common (50%) and pyloric gland subtype (simple mucinous and complex‐nonmucinous) in 20% of the cases, with only one of the simple mucinous polyps showing high-grade dysplasia. The least frequent subtype was intestinal, representing 8% of the cases. There was a significant difference in the risk of invasion among the subtypes, with the biliary subtype showing a stronger association with invasive carcinoma compared to the pyloric gland subtype. In the following, pyloric gland subtype, the most frequently encountered in the clinical practice, is discussed in detail [3].

## 2. Histopathogenesis

Constant inflammation and irritation of the epithelium in chronic cholecystitis may lead to many gross and microscopic changes including metaplasia. The most common metaplastic change in the gallbladder epithelium (reported in 50% of cholecystectomies) [6] is gastric metaplasia. It may take the following forms: (1) gastric foveolar-type epithelium replacing the biliary-type epithelium and (2) formation of pyloric-like glandular structures generally in the lamina propria, recapitulating antral pyloric glands or duodenal Brunner's glands. These pyloric-like glands have a lobular architecture and are lined by cuboidal or columnar cells with basal nuclei and smooth cytoplasm. However, if the chief cells or parietal cells are also present, the changes are most likely gastric heterotopia rather than metaplasia. In most of the daily routine cases, these glands are far and few between. However, if the changes become exuberant, they can form polypoid lesions, whether they are neoplastic or reactive in nature, is still under debate since many studies have included subcentimeter lesions in their cohorts [7]. Adsay and coauthors considered the less than 1 cm lesions to be polypoid metaplasia or benign fibromyoglandular polyps if the increased stroma or splays of smooth muscle had separated the glands [3].

## 3. Macroscopic and Microscopic Features

Pyloric gland adenomas are soft-tan excrescences which have a thin stalk that is readily detached from the surface. If the prosector is not aware of a possible polyp, the detached lesion could be mistaken as biliary sludge or debris mixed with the thick luminal contents and sampled in the second round of gross evaluation by searching the specimen container.

**Figure 1:**
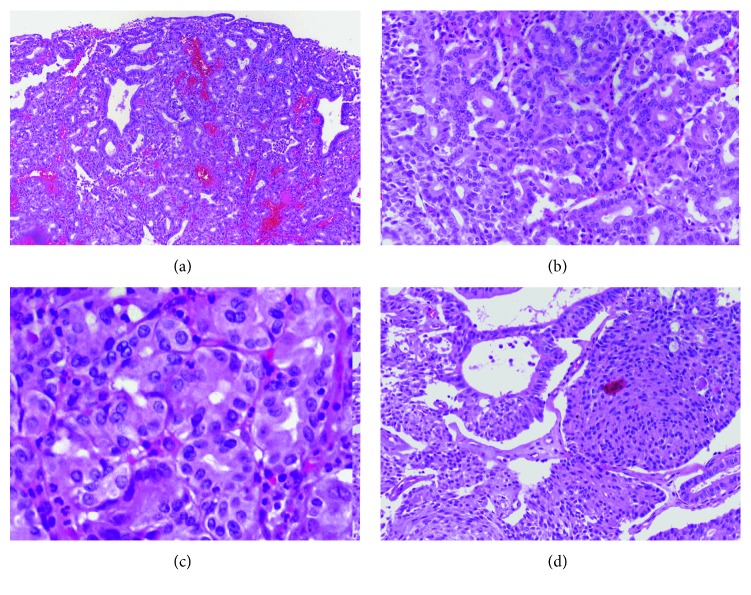
(a) Polypoid lesion with tubuloglandular proliferation. (b) Small packed and round pyloric glands with little intervening stroma. (c) The cells have a low nuclear-cytoplasmic ratio with small and smooth-border nuclei; some have optic clearing corresponding to biotin-rich nuclei. (d) Squamoid morules are syncytium of bland meningothelial-like, nonkeratinizing spindle cells with a whorling pattern. They lack discernible intercellular bridges, unlike real squamous metaplasia (hematoxylin-eosin stain, original magnification 40x (a), 100x (b), 400x (c), and 100x (d)).

Pyloric gland adenomas are characterized by packed, small, round, and uniform pyloric glands. They have a tubular configuration and low nuclear-cytoplasmic ratio with little or no intervening stroma (Figures 1(a)–1(c)) [2, 8]. Adsay et al. have introduced a distinct subgroup within the pyloric gland adenomas called complex nonmucinous. They are highlighted by a complex growth of small tubular units that diffusely express pyloric gland immunomarker, MUC6. These showed a more irregular, variegated, and cystically dilated glandular units. The nuclear-cytoplasmic ratio is frequently higher than the simple group, and nucleoli are often evident [3]. Paneth and neuroendocrine cells with focal hyalinization of the stroma could be identified in a subset of cases [3]. There is also a morphologic variant of pyloric gland polyps which recapitulates Brunner's gland-like morphology with tall apical mucin and peripherally located small nuclei. Squamoid morules (also known as spindle cell metaplasia) are a syncytium of bland meningothelial-like nonkeratinizing spindle cells with a whorling pattern (Figure 1(d)). They lack discrete cytoplasmic borders, real keratinization or discernible intercellular bridges, unlike real squamous metaplasia which is a rare finding in gallbladders. They have occasional optically clear, biotin-rich nuclei which are highlighted by nuclear expression of beta-catenin [9, 10]. Squamoid morules are specific to pyloric gland adenomas and are not seen in other subtypes of ICPN. They are reported in 10% to 64% of pyloric gland adenomas in different studies [3, 4, 6, 11].

## 4. Immunohistochemical Features

**Figure 2:**
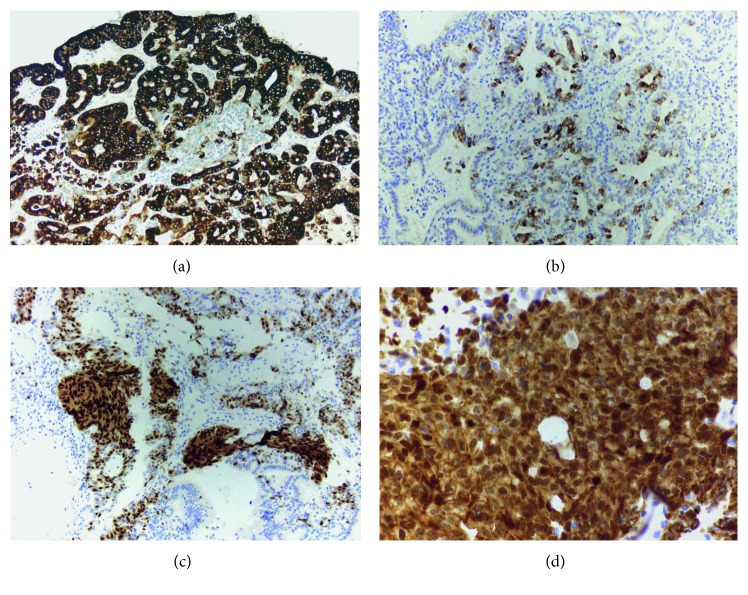
(a) Pyloric gland adenoma with a diffuse expression of MUC6. (b) Focal positivity with MUC5AC in pyloric gland adenoma, reiterating mixed-lineage nature of ICPN of the gallbladder. (c) Aberrant CDX2 expression in squamoid morules of pyloric gland adenoma. (d) Nuclear expression of beta-catenin in squamoid morules (immunohistochemical stain, original magnification 100x (a through c) and 600x (d)).

Pyloric gland adenomas are highlighted by the diffuse cytoplasmic positivity of MUC6, a pyloric marker (Figure 2(a)). However, sometimes they can show focal staining with MUC5A (a foveolar marker, positive in gastric foveolar-type adenomas, Figure 2(b)) and MUC1 (a pancreatobiliary marker, positive in biliary-type adenomas).

Areas of high-grade dysplasia in many types of ICPN might show positivity for MUC1 suggesting its potential for detection of high-grade dysplasia. These findings overall emphasize that ICPNs might exhibit a spectrum of cell lineages. However, the type and prognosis are driven by the dominant (over 75%) component [3].

CDX2 is a marker of intestinal differentiation in many organs including pancreas, urinary bladder, and ovary [12, 13]. In gallbladder, CDX2 highlights areas of intestinal metaplasia due to chronic cholecystitis and cholelithiasis and is associated with MUC2 expression [2, 3]. However, pyloric gland adenomas are an exception to this rule, as they show aberrant CDX2 expression in squamoid morules (Figure 2(c)), which is the result of nuclear expression of beta-catenin (Figure 2(d)) [14], described in squamous morules of endometrial endometrioid adenocarcinomas [15].

## 5. Prognosis

The overall survival after diagnosis of ICPN is high. Patients with ICPNs not associated with invasive adenocarcinomas, regardless of the subtype, had a 1-, 3-, and 5-year survival rates of 90%, 90%, and 78%, respectively, in Adsay and coworkers' study [3]. Among ICPNs, pyloric gland subtype had the best prognosis as it showed the lowest risk of associated invasive carcinoma (only in 18% of cases [3]). The presence of high-grade dysplasia, biliary, or foveolar subtype and papillary formation were considered significant histologic risk factors associated with carcinogenesis [3].

Albeit histologic classification of ICPNs is not widely used, it would help the pathologist to determine the risk of concurrent invasive carcinoma in the gallbladder and to direct the extent of sampling of the grossly uninvolved gallbladder. The presence and extent of high-grade dysplasia needs to be reported as it has direct association with prognosis.

## 6. Treatment

Most of the gallbladder polyps are asymptomatic unless they are large, multiple, or detached from the mucosa which results in free-floating fragments that can cause biliary colic. Thus, most of the polyps are diagnosed during imaging procedures performed for other reasons.

One of the well-established predictors of malignancy and a surgical management criterion in gallbladder polyps is the size of over 10 mm [16–19]. Conversely, some have proposed the higher cutoff value of 15 mm for surgical interventions [20]. Many authorities recommend noninvasive management for the polyps smaller than 10 mm [21–24] even though reports of malignancy in smaller-sized polyps exist as well [25, 26].

Overall, the accepted criteria for cholecystectomy in asymptomatic patients with polyps include (I) older than 50 years old, (II) polyps of over 10 mm, (III) concurrent gallstones, or (IV) continuous growth of the polyp in the follow-up imaging studies [5].
